# Larvae act as a transient transmission hub for the prevalent bumblebee parasite *Crithidia bombi*

**DOI:** 10.1016/j.jip.2017.06.001

**Published:** 2017-09

**Authors:** Arran J. Folly, Hauke Koch, Philip C. Stevenson, Mark J.F. Brown

**Affiliations:** a Royal Holloway University of London, Egham, UK; b Royal Botanic Gardens, Kew, UK; c Natural Resources Institute, University of Greenwich, Kent, UK

**Keywords:** *Bombus terrestris*, Trypanosomatid, Epidemiology, Brood diseases

## Abstract

Disease transmission networks are key for understanding parasite epidemiology. Within the social insects, structured contact networks have been suggested to limit the spread of diseases to vulnerable members of their society, such as the queen or brood. However, even these complex social structures do not provide complete protection, as some diseases, which are transmitted by workers during brood care, can still infect the brood. Given the high rate of feeding interactions that occur in a social insect colony, larvae may act as disease transmission hubs. Here we use the bumblebee *Bombus terrestris* and its parasite *Crithidia bombi* to determine the role of brood in bumblebee disease transmission networks. Larvae that were artificially inoculated with *C. bombi* showed no signs of infection seven days after inoculation. However, larvae that received either an artificial inoculation or a contaminated feed from brood-caring workers were able to transmit the parasite to naive workers. These results suggest that the developing brood is a potential route of intracolonial disease transmission and should be included when considering social insect disease transmission networks.

## Highlights

• *Bombus terrestris* larvae showed no signs of *Crithidia bombi* infection.
• *B. terrestris* larvae were able to transmit the parasite to naive workers.
• Brood-worker interactions may be a component of social insect disease networks.

## Graphical abstract

**Figure:**
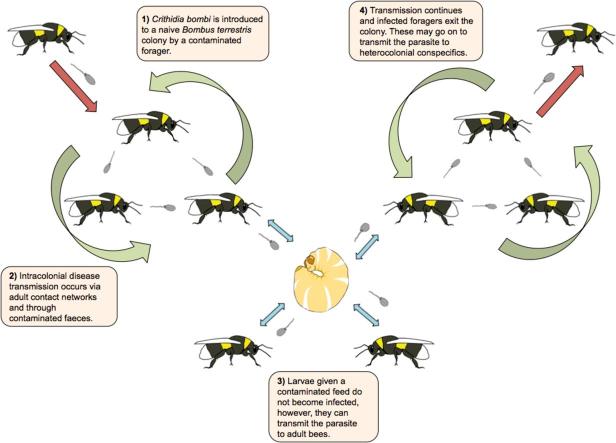

## 1 Introduction

To be successful, a parasite must have effective host transmission and the ability to maintain parasitaemia once infection is established (Price, 1980). Understanding the epidemiology of parasites is consequently key for elucidating host-parasite interactions. The high population densities and low genetic variability of social insects may provide an ideal environment for pathogen transmission (Schmid-Hempel, 1998, but see Van Baalen and Beekman, 2006). Consequently, on top of individual immune mechanisms, some insect societies have evolved ‘social immunity’ (reviewed by Cremer et al., 2007). One potential mechanism of social immunity is the evolution or co-option of structured contact networks among individuals to minimize the spread of disease (Naug, 2008, Naug and Smith, 2007, Schmid-Hempel, 1998, Schmid-Hempel and Schmid-Hempel, 1993). Such networks, e.g., centrifugal polyethism in ants (Hölldober and Wilson, 1990, Schmid-Hempel, 1998) and heterogeneous interaction networks in honeybees (Naug, 2008, Naug and Camazine, 2002), may limit the spread of parasites within colonies, and give specific protection to the queen and brood, which are essential to the reproductive success of the colony. Nevertheless, even in large, complex insect societies, such protection is incomplete (Bailey, 1956, reviewed by Bailey and Ball, 1991, de Miranda and Genersch, 2010, Hansen and Brødsgaard, 1999). One reason for this is the high rate of worker-brood feeding interactions that are required for successful larval development. Consequently, in contrast to the prevailing view, brood may have the potential to act as a transmission hub in social insect colonies.

Bumblebees and their parasite, *Crithidia bombi*, provide an excellent model system to address this question. Bumblebees exist as small, relatively simple eusocial colonies (Wilson, 1971). Most colonies acquire *Crithidia bombi* from transmission through foraging outside of the nest (Imhoof and Schmid-Hempel, 1999, Jones and Brown, 2014, Shykoff and Schmid-Hempel, 1991), presumably from flowers that have been contaminated by a visiting infected worker (Durrer and Schmid-Hempel, 1994, Ruiz-González et al., 2012). The parasite is transferred to other workers in the colony through contact networks (Otterstatter and Thomson, 2007, Shykoff and Schmid-Hempel, 1991). However, how these networks relate to the colony’s brood is unclear. Currently, it remains unknown whether *C. bombi* is infective towards bumblebee larval stages, and thus whether bumblebee brood are integral to intra-colonial transmission networks. However, other insect trypanosomatids infect both adults and larvae (e.g., Hamilton et al., 2015). In addition, even in the absence of infection, the repeated oral interactions (termed “trophallaxis”, Wilson, 1971) between larvae and adult workers could provide the opportunity for brood to act as a transient hub for parasite transmission. Here we ask the following questions: (1) can *B. terrestris* larvae become infected with the gut trypanosome *C. bombi*?, and (2) can larvae act as a source of infection for workers?

## 2 Materials and methods

### 2.1 Colony origin

Four *B. terrestris* colonies (hereafter referred to as colonies A, B, C & D) (containing a queen, brood and a mean of 95 (±6.1 S.E.) workers) were obtained from Biobest, Belgium. Colonies were kept in a dark room at 25 °C and 55% humidity (red light was used for colony manipulation). To ensure colonies were healthy and developing normally they were monitored for seven days prior to use in the experiments described below.

### 2.2 Testing whether bumblebee larvae can be infected by *Crithidia bombi.*

To create a parasite source, wild *B. terrestris* queens, naturally infected with *C. bombi*, were collected from Windsor Great Park, UK (SU992703), in the spring of 2016. The faeces of these bees were mixed with sugar water (1:1) and fed orally to ten workers removed from Colony D. The inoculated workers were returned to colony D, enabling parasite transmission to occur and creating a stock population for *C. bombi* acquisition. This stock population was maintained under dark room conditions (see above) and fed *ad libitum* pollen and sugar water.

A *C. bombi* inoculant was prepared by combining the faeces of twenty stock bees, which was then diluted in 1 ml of 0.9% insect Ringer solution. To purify the *C. bombi* inoculant a modified version of the Cole (1970) triangulation protocol was used. The *C. bombi* cells in the resulting solution were counted using a Neubauer haemocytometer and the concentration of cells was calculated at 3800/μl.

From the remaining three colonies (A, B & C) forty-two workers per colony were randomly selected and placed into individual quarantine. Quarantine chambers (n = 126) comprised a 16 × 10 × 8 cm plastic box. The lid was modified to allow the insertion of a 10 ml tube to provide *ad libitum* sugar water, and each quarantine box was also provided with 0.1 g of pollen. All quarantined bees were monitored for seven days. This period of time enabled reliable detection of already existing *C. bombi* infections (Logan et al., 2005, Schmid-Hempel and Schmid-Hempel, 1993). All workers were then screened for the common parasites, *C. bombi, Nosema* spp*.* and *Apicystis bombi*, by microscopic examination of faecal samples using a phase contrast microscope at 400× magnification. No infections were identified in any workers at this stage. These quarantined workers were used for all further experimental procedures to ensure there was no external parasite source.

Once workers had passed successfully through quarantine, ten larvae from each of the remaining three colonies were randomly assigned across experimental or control micro-colonies (1 each per colony). Each micro-colony (n = 6) was housed in a 14 × 8 × 5.5 cm acrylic box and contained brood casing with five larvae at 2nd/3rd instar. Prior to inoculation these were kept without access to food or workers, under dark room conditions (outlined above) for one hour to ensure a feeding response. To inoculate larvae, sugar water and pollen were first combined (3:1) to create an artificial worker feed. This was then combined in equal proportions (100 μl:100 μl) with the *C. bombi* inoculant (see above) to create a master-mix. Experimental larvae were exposed by opening the brood casing, and each was then fed 6 μl of inoculated feed containing 11,400 parasite cells using a 20 μl micropipette. Each control larva was fed 6 μl of sugar water and pollen (3:1) in a similar fashion. Larvae were left to consume the entire inoculant until no trace was visible under a stereomicroscope (20× magnification). Post inoculation the brood casing was resealed manually and larvae were returned to their micro-colonies. Each micro-colony was then given three quarantined workers to provide brood care, and was provisioned with *ad libitum* pollen and sugar water. After seven days, larvae were removed and their gut was isolated by dissection. The gut was homogenized in 0.5 ml of 0.9% insect Ringer solution and screened for *C. bombi* by microscopic examination using a phase contrast microscope (400× magnification). Workers from each micro-colony were also screened for *C. bombi* using microscopic examination of faecal samples*.* If an infection was identified a Neubauer haemocytometer was used to calculate an average cell count.

### 2.3 Investigating whether larvae can act as a transmission hub for *Crithidia bombi*

Workers from each experimental micro-colony described above were found to have *C. bombi* infections. To investigate if this transmission had occurred during trophallaxis or via the inoculant residue left on the larvae a further experiment was designed. As before ten larvae were removed from each of the three donor commercial colonies (A, B & C). Experimental and control micro-colonies (n = 6) each containing five larvae at 2nd/3rd instar were then set up. Larvae were inoculated as before, however once larvae had consumed the inoculum they were removed from their brood casing and submerged in 15 ml of ddH_2_O and dried using a paper towel. Larvae were placed back in their brood casing which was resealed and returned to their respective micro-colonies with three quarantined workers to provide brood care, and were provisioned with *ad libitum* pollen and sugar water. After a period of seven days workers were removed and screened for *C. bombi* infection via microscopic examination of faeces. Again, under these conditions, workers were found to have *C. bombi* infections. A final, more conservative iteration of this methodology using the same sample size was undertaken where the cleaning process was repeated twice per larvae post inoculation. Workers were screened as before after a period of seven days.

As larval cleaning (see above) is not representative of an ecologically relevant scenario, to determine if larvae acted as a pathogen hub after receiving a parasite-contaminated feed, a serial transfer experiment was used. Again ten larvae were removed from each of the three donor commercial colonies (A, B & C). Experimental and control micro-colonies (n = 6) each containing five larvae at 2nd/3rd instar were then set up. To ensure experimental manipulation was not the cause of *C. bombi* transmission, inoculation was undertaken by three workers per micro-colony (hereafter called “nurse cohort 0”), by providing them with a food source contaminated with *C. bombi* to feed to experimental larvae. A *C. bombi* inoculant, as described above, was mixed with artificial worker feed in a 1:5 ratio (to make up 1 ml), which nurse cohort 0 were allowed to feed to experimental larvae for a period of 24 h. Control groups were similarly fed a pollen sugar water mix. At the end of the 24-h period the brood casing containing all larvae was removed and placed into a sterile micro-colony box with three newly quarantined workers (hereafter called “nurse cohort 1”) and provided *ad libitum* pollen and sugar water. After 24 h exposure nurse cohort 1 were removed and quarantined for seven days before screening for *C. bombi* (as described earlier). Larvae were simultaneously transferred into a new sterile micro-colony box and provisioned with three newly quarantined workers (hereafter called “nurse cohort 2”) and *ad libitum* pollen and sugar water. This serial transfer continued for a total of three days post inoculation, with all workers being screened for *C. bombi* infections seven days after exposure to the contaminated larvae. If infection was observed a haemocytometer was used to quantify the parasite load. In all experiments infection intensities were compared with ANOVA tests using R programming language (R Core Team, 2016). All graphical outputs were undertaken in R, using ggplot 2 (Wickham, 2009).

## 3 Results

### 3.1 Can *Crithidia bombi* infect Bombus *terrestris* larvae?

All *B. terrestris* larvae (n = 15) across three micro-colonies showed no signs of *C. bombi* infection seven days after inoculation. However transmission of the parasite from the larvae to the workers occurred in all artificially inoculated micro-colonies with 100% of workers becoming infected. There were no statistical differences in worker infection intensity across the micro-colonies (*F*_2,6_ = 3.35, *P* = 0.1). Neither larvae nor workers from the control replicates showed any sign of infection, as expected.

### 3.2 Can larvae act as a transmission hub for *Crithidia bombi*?

**Fig. 1:**
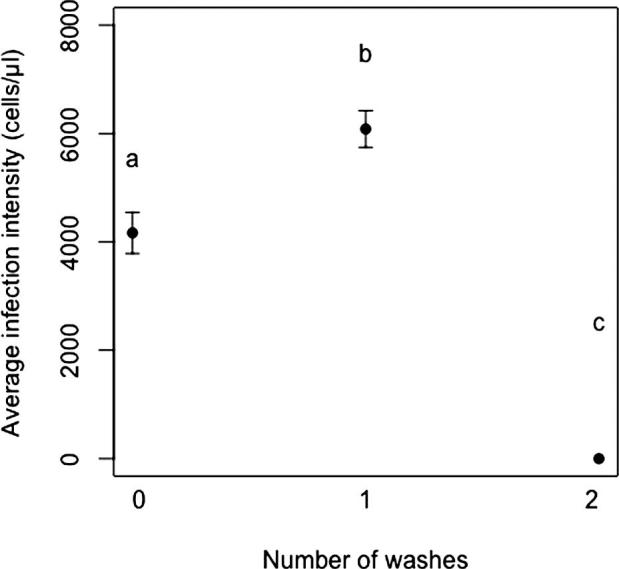
*C. bombi* infection intensity in *B. terrestris* workers (mean ± SEM) seven days after exposure to artificially inoculated larvae that underwent different washing regimes. X-axis denotes number of experimental washes. Statistical differences between treatments are represented with letters (*F*_2,26_ = 111.44, *P* = <0*.*001).

In the first experiment, where larvae were washed once after experimental inoculation, all nine workers that were exposed to these larvae became infected by *C. bombi.* There was no statistical difference across micro-colonies in the infection intensities of workers after larval washing (*F*_2,6_ = 1.82, *P* = 0.24). None of the workers in the control colonies became infected, as expected. In the second experiment, where larvae were washed twice after inoculation, no exposed workers developed a *C. bombi* infection. Including the first experiment with no larval cleaning, infection intensities were significantly different across all three washing treatments (*F*_2,26_ = 111.44, *P* = <0.001) (Fig. 1).

**Fig. 2:**
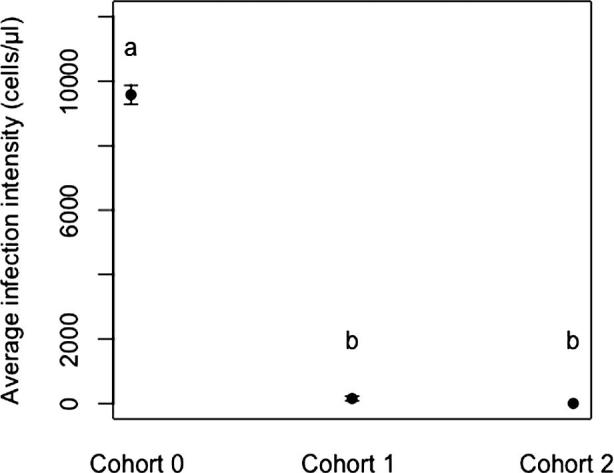
*C. bombi* infection intensity in *B. terrestris* workers from three nurse cohorts of the serial transfer experiment (mean ± SEM) seven days after exposure to inoculated larvae. Statistical differences between nurse cohorts are represented with letters (*F*_2_*_,_*_26_ = 977*.*57, *P* =  < 0*.*001).

In the serial-transfer experiment, nurse cohort 0 that were used to inoculate larvae prior to the serial transfer of brood were quarantined for seven days before being screened, and all tested positive for *C. bombi* infection. Again, infection intensities did not differ significantly among micro-colonies (*F*_2,6_ = 3.81, *P* = 0.09). These workers had come into direct contact with the *C. bombi* inoculant while feeding larvae, and screening them confirmed that the inoculum was infective to workers. A third of quarantined bees (n = 9) from the nurse cohort 1 developed *C. bombi* infections, although infection intensities were 100-fold lower compared to nurse cohort 0, and no infections were identified in bees from nurse cohort 2. Due to the number of bees infected after the first serial transfer, it was not possible to analyze infection intensity across micro-colonies. However, infection intensities in nurse cohort 1 and 2 were significantly lower than in nurse cohort 0 (*F*_2,26_ = 977.57, *P* = <0.001) (Fig. 2).

## 4 Discussion

Here we have shown that under laboratory conditions *B. terrestris* larvae had no signs of *C. bombi* infection seven days after inoculation. However, the feeding interaction between workers and larvae facilitated the transmission of *C. bombi*, with the larvae acting as a transient parasite transmission hub.

Microbial parasites and their invertebrate hosts have been co-evolving in an ecological arms race to either maintain parasitaemia or to reduce parasite loads respectively (Anderson and May, 1981, Cremer et al., 2007, Price, 1980, Schmid-Hempel, 1998). Given that other insect trypanosomatids can infect both adults and larvae (Hamilton et al., 2015), and given the likely frequent contact between bumblebee larvae and *C. bombi*, it is surprising that the parasite has not evolved to exploit these additional hosts. However, in high sugar, aqueous environments the ability of *C. bombi* to persist is drastically reduced (Cisarovsky and Schmid-Hempel, 2014) and this may explain the inability of *C. bombi* to infect bumblebee larvae. Bumblebee larval guts are likely to have high osmotic potential due to the sugar heavy feeding regime they receive during broodcare (Pereboom, 2000). Whilst larvae must consume and metabolize sugars for growth and development they do not defecate and therefore it is likely that the high osmotic potential in their gut undergoes less reduction than might be expected in adult workers which regularly defecate. Differences in gut conditions between the two bumblebee life stages may therefore explain why only adults support *C. bombi* colonization and subsequent infection.

Previous work has highlighted that contact networks between adults are a significant predictor of *C. bombi* infection risk (Otterstatter and Thomson, 2007). Our results suggest that larvae should also be integrated into bumblebee disease contact networks. In adult bumblebees *C. bombi* transmission rate is high as the infection is spread via contaminated faeces (Schmid-Hempel, 1998). Our results, combined with the high frequency of feeding interactions that occur in a bumblebee nest (Katayama, 1973), suggest that the parasite is able to indirectly use larvae as a transmission hub to infect naive adult hosts. At a more general level, bumblebees are not noted for their hygienic nest conditions (Wilson, 1971) and therefore trophallactic interactions between workers and larvae are likely to be contaminated with an array of parasites. Consequently, larval transmission hubs could be relevant for other bumblebee parasites that are dependent on contact networks (Fürst et al., 2014, Rutrecht and Brown, 2008), particularly given the large proportion of the colony workforce that is involved in brood care (Pendrel and Plowright, 1981). Thus, we suggest that adult-brood trophallactic transmission is likely to be a component of social insect disease networks generally.

When larvae received either zero or one wash post inoculation, workers providing brood care went on to develop *C. bombi* infections that are consistent with previously reported infection intensities (Logan et al., 2005, Schmid-Hempel and Schmid-Hempel, 1993). Interestingly, when larvae were washed twice post inoculation, brood caring workers did not become infected with *C. bombi*. The rationale behind larval cleaning was to provide evidence that transmission of parasites occurred during trophallaxis and not from inoculant residue left on the larvae. Our data show a non-linear response across washing treatments. One explanation for this is that submerging larvae in ddH_2_O would be an unnatural stress that may cause larvae to open their buccal cavity, consequently washing out any residual parasites. It is likely that two washes effectively removed all parasite cells from both the larval surface and the buccal cavity. In contrast, in the one wash treatment, our worker infection results show that not all parasite cells were removed.

Following inoculation of larvae by nurse cohort 0, disease establishment only occurred in nurse cohort 1 in the serial transfer experiment, demonstrating the transient nature of transmission via brood-worker interactions. However, we would note that, under natural conditions, brood are likely being repeatedly exposed to the parasite through feeding interactions, and thus whilst transmission is transient after a single feed, it is likely to be continuous under natural conditions. We acknowledge that bumblebee parasite transmission may occur on surfaces (Durrer and Schmid-Hempel, 1994, Ruiz-González et al., 2012) and that brood casing may facilitate this. However, brood-caring workers are primarily focused on direct contact with developing larvae and this would be the exchange point for contaminated food. In addition, experimentally cleaned larvae were able to transmit the parasite to naïve workers suggesting that disease transmission is likely to be occurring during trophallaxis, rather than through contact with brood casing. Nurse cohort 0, which were exposed directly (through feeding the larvae) to the parasite source during the serial transfer experiment went on to develop *C. bomb*i infections that are consistent with previously reported infection intensities (Logan et al., 2005, Schmid-Hempel and Schmid-Hempel, 1993). Parasitaemia in workers infected during the experimental cleaning methodology was also consistent with previously reported infection intensities. Interestingly however, even after an incubation period of seven days the parasite loads of infected workers from nurse cohort 1 were a 100-fold lower than what has been reported in *C. bombi* parasitaemia (Logan et al., 2005). Previous work has shown that a dose of 5000 cells is enough to lead to infection (Brown et al., 2003), but how dose relates to subsequent infection intensity remains unknown. One explanation for the low infection intensities in the serial transfer experiment may therefore be that they were initiated by significantly fewer parasite cells. It seems unlikely that these lower parasitaemias are due to differences in individual susceptibility (Barribeau and Schmid-Hempel, 2013, Brunner et al., 2013, Sadd and Barribeau, 2013) particularly as the workers that exhibited low infection intensities were a random sample from the same colonies as the first round of workers that showed much higher infection intensities.

Bumblebee parasite prevalence is increasing (Cameron et al., 2016, Schmid-Hempel et al., 2014) and this has been linked to bumblebee population declines (Cameron et al., 2011). More specifically, the global prevalence of *C. bombi* is rising and this has been linked to the transportation of commercial bumblebee colonies for pollination services (Graystock et al., 2013, Whitehorn et al., 2013). *C. bombi* has significant impacts on queen fitness (Brown et al., 2003), high transmission potential (Otterstatter and Thomson, 2007, Schmid-Hempel and Schmid-Hempel, 1993), and high prevalence (Jones and Brown, 2014, Shykoff and Schmid-Hempel, 1991). Here we have identified that bumblebee larvae can act as an intracolonial transmission hub for *C. bombi*. We believe that these results enhance our understanding of the transmission potential of *C. bombi* and help to explain how it maintains such a high environmental prevalence.

## Authorship statement

AF, HK, PS & MB devised the experiment. AF, HK & MB designed the experiments. AF was responsible for experimental work and writing the manuscript. HK, PS & MB provided feedback on the manuscript.
